# Dimethyl Sulfoxide Enhances Kaposi’s Sarcoma-Associated Herpesvirus Production During Lytic Replication

**DOI:** 10.3389/fmicb.2021.778525

**Published:** 2021-12-16

**Authors:** Su-Kyung Kang, Myung-Ju Lee, Ho-Hyun Ryu, Jisu Lee, Myung-Shin Lee

**Affiliations:** ^1^Department of Microbiology and Immunology, Eulji University School of Medicine, Daejeon, South Korea; ^2^Eulji Biomedical Science Research Institute, Eulji University School of Medicine, Daejeon, South Korea

**Keywords:** KSHV, DMSO, herpesvirus, lytic replication, viral production

## Abstract

Kaposi’s sarcoma-associated herpesvirus (KSHV) is an etiologic agent of Kaposi’s sarcoma, primary effusion lymphoma, and multicentric Castleman disease. In studies of KSHV, efficient virus production and isolation are essential. Reactivation of KSHV can be initiated by treating latently infected cells with chemicals, such as 12-O-tetradecanoyl-phorbol-13-acetate and sodium butyrate. These chemicals have been used as tools to induce lytic replication and viral production in KSHV-producing cell lines. Dimethyl sulfoxide (DMSO) is an organosulfur compound that is frequently used as an aprotic solvent similar to water. In experiments exploring signaling pathways in KSHV-infected cells, DMSO treatment alone as a vehicle affected the lytic gene expression of KSHV. However, to the best of our knowledge, the effects of DMSO on KSHV-producing cells have not yet been reported. Therefore, in this study, we investigated whether DMSO could be used as a reagent to enhance viral production during lytic replication in KSHV-producing cells and assessed the underlying mechanisms. The effects of DMSO on KSHV production were analyzed in iSLK BAC16 cells, which have been widely used for recombinant KSHV production. We found that the production of KSHV virions was significantly increased by treatment with DMSO during the induction of lytic replication. Mechanistically, lytic genes of KSHV were enhanced by DMSO treatment, which was correlated with virion production. Additionally, DMSO induced the phosphorylation of JNK during lytic replication, and inhibition of JNK abolished the effects of DMSO on lytic replication and virion production. Our findings showed that additional treatment with DMSO during the induction of lytic replication significantly improved the yield of KSHV production.

## Introduction

Kaposi’s sarcoma-associated herpesvirus (KSHV) is a gammaherpesvirus belonging to the family Herpesviridae (Chang et al., 1994). Kaposi’s sarcoma (Boshoff and Weiss, 2002), multicentric Castleman disease (Powles et al., 2009), primary effusion lymphoma (Cesarman et al., 1995), and inflammatory cytokine syndrome are known to be associated with KSHV (Polizzotto et al., 2016). The isolation of virions is a necessary process for studying KSHV. Similar to other herpesviruses, the life cycle of KSHV consists of two phases: latent and lytic replication cycles (Yan et al., 2019). Virions are produced only during lytic replication. Therefore, efforts have been made to extract large amounts of virus by effectively inducing lytic replication from KSHV-harboring cells, and various chemicals have been tested. 12-O-Tetradecanoyl-phorbol-13-acetate induces lytic replication of KSHV by activating the mitogen-activated protein kinase (MAPK)/extracellular signal-regulated kinase (ERK) pathway (Cohen et al., 2006). Sodium butyrate activates the KSHV ORF50 promoter by inducing histone H3 and H4 hyperacetylation (Lu et al., 2003). Additionally, inomycin (Chang et al., 2000), epinephrine/norepinephrine (Chang et al., 2005), and valproic acid are potent inducers of KSHV reactivation (Shin et al., 2014).

Dimethyl sulfoxide (DMSO) is a polar aprotic molecule with a strong affinity for water. DMSO exhibits a rapid penetration effect and enhances the penetration of other substances. Moreover, the low systemic toxicity of DMSO enables its wide application as a solvent (Brayton, 1986). DMSO has been shown to have various effects on human cellular processes and the epigenetic landscape, such as DNA methylation, mRNA and protein expression, and microRNA expression (Verheijen et al., 2019). In several enveloped viruses, such as influenza A virus, Newcastle disease virus, and Semliki Forest virus, DMSO has a stimulating effect on virus yield (Scholtissek and Muller, 1988). Furthermore, previous studies have demonstrated that DMSO can affect the human herpesvirus life cycle. In herpes simplex virus-1, DMSO blocks productive infection by inhibiting viral DNA replication and viral mRNA expression (Aguilar et al., 2002). In human cytomegalovirus, DMSO enhances virion production in virus-infected cells (Tanaka et al., 1985; West et al., 1988). However, no studies have investigated the effects of DMSO on KSHV.

In our *in vitro* experiments with KSHV, we found that KSHV-harboring cells treated with DMSO as a control vehicle for chemicals showed alterations in lytic gene expression. Therefore, we wondered whether DMSO may have positive effects on KSHV production in KSHV-producing cells. Accordingly, in this study, we investigated whether DMSO could be used as an enhancing agent for the lytic replication and production of KSHV. Our results revealed that DMSO enhanced KSHV production in iSLK BAC16 cells during induced lytic replication through increased phosphorylation of c-Jun N-terminal kinase (JNK), suggesting that DMSO could be an effective enhancing agent for KHSV production.

## Materials and Methods

### Cell Culture and Reagents

iSLK BAC16 cells were cultured in Dulbecco’s Modified Eagle’s Medium (DMEM)/high glucose (Welgene, Gyeongsan, South Korea) with 10% fetal bovine serum (FBS; GenDEPOT, Katy, TX, United States) and 1% antibiotic-antimycotic (Invitrogen, Waltham, MA, United States). Hygromycin B (1.2 mg/mL; Invitrogen), geneticin (250 μg/mL; Invitrogen), and puromycin (1 μg/mL; Invitrogen) were added and cultured to maintain the latent infection of iSLK BAC16. Human umbilical vein endothelial cells (HUVECs) were purchased from PromoCell (Heidelberg, Germany) and cultured in endothelial cell growth medium 2 (PromoCell) containing cell growth supplements. BCBL-1 cells were cultured in RPMI 1640 (Biowest, Riverside, MO, United States) supplemented with 10% FBS. All cells were cultured at 37°C in a humidified atmosphere containing 95% air and 5% CO_2_. SP600125 was purchased from Calbiochem (San Diego, CA, United States). DMSO was purchased from LPS Solution (Daejeon, South Korea).

### Virus Isolation and Infection

iSLK BAC16 cells harboring recombinant KSHV BAC16 were used to produce virions (Brulois et al., 2012). iSLK BAC16 cells were treated with 1.2 mM sodium butyrate (Sigma, Burlington, MA, United States) and 50 μg/mL doxycycline (Sigma) for 48 h to induce lytic replication. Upon induction of lytic replication, DMSO was added into culture media together with sodium butyrate and doxycycline at 0.1%, 0.5%, or 1% of the total volume. For virus isolation, the culture medium was collected and centrifuged at 300 × *g* for 10 min at 4°C to remove cell debris from the culture supernatant. The supernatant was again centrifuged at 2,000 × *g* for 10 min at 4°C, and the supernatant was collected. The supernatant was collected and centrifuged at 100,000 × *g* for 1 h at 4°C. The virus pellet was resuspended in cold phosphate-buffered saline (PBS) and stored at –80°C until the virus stock was used. KSHV infection was performed as described previously (Kang et al., 2021). Briefly, the prepared KSHV stock was added to Gibco Opti-MEM (Invitrogen) containing 5 μg/mL polybrene (Santa Cruz Biotechnology, Santa Cruz, CA, United States). HUVECs were seeded in 6-well culture plates the day before KSHV infection. KSHV infection was performed by centrifugation at 2,600 rpm for 1 h at 25°C. After centrifugation, the medium was changed to endothelial cell growth medium 2 (PromoCell), and HUVECs were then incubated overnight at 37°C in a humidified atmosphere containing 5% CO_2_.

### Flow Cytometry Analysis of Kaposi’s Sarcoma-Associated Herpesvirus Infectivity

Human umbilical vein endothelial cells infected with KSHV BAC16 were detached from culture wells with trypsin-ethylenediaminetetraacetic acid (EDTA) solution (Invitrogen) and neutralized using a culture medium. After washing with 1 × PBS, the cells were resuspended in PBS containing 1% FBS. Because cells infected with the recombinant KSHV BAC16 expressed green fluorescence protein (GFP), infected cells were analyzed using a Guava easyCyte flow cytometer (Luminex Corporation, Austin, TX, United States) and InCyte 3.1 software (Luminex Corporation).

### Western Blotting

Lysis buffer (20 mM Tris-HCl, 150 mM NaCl, 10 mM EDTA, 1 mM EGTA, and 1% Triton X-100) was prepared for cell lysates. To inhibit endogenous and exogenous protease and phosphatase inhibitors, 0.1 mM sodium fluoride, 0.1 mM phenylmethylsulfonyl fluoride, 1 mM beta-glycerophosphate, and 0.1 mM sodium orthovanadate were added to the lysis buffer immediately before cell lysis. The cell lysate was incubated on ice for 30 min and then centrifuged at 13,000 rpm for 10 min at 4°C. The supernatants were collected, and the protein concentrations were measured using a bicinchoninic acid assay (Pierce, Rockford, IL, United States). Proteins were separated by sodium dodecyl sulfate polyacrylamide gel electrophoresis on 10% or 12% gels and transferred to nitrocellulose membranes. The membranes were blocked overnight at 4°C with TBST (1 × Tris-buffered saline containing 0.1% Tween 20) containing 5% skim milk. The primary antibody was diluted in 5% bovine serum albumin/TBST and reacted with the sample by rocking at 4°C overnight. The secondary antibody diluted with TBST containing 5% skim milk was reacted on a rocker for 1 h at room temperature. Immunolabeled proteins were detected using Clarity Western ECL Substrate (Bio-Rad Laboratories, Hercules, CA, United States) and an Amersham ImageQuant 800 biomolecular imager (GE Healthcare, Chicago, IL, United States). Immunodetection was performed using rabbit antibodies against HHV8 ORF50 (Bioss, Woburn, MA, United States, bs-0860R), total AKT (Bioss, bs-5050R), total p38 MAPK (Bioss, bs-50503R), phosphorylated p38 MAPK (Thr180/Tyr182, Bioss, bs-50486R), or phosphorylated ERK (Thr202/Tyr204, Bioss, bs-50534R); mouse antibodies against HHV-8 K8.1A/B (sc-65446), JNK (total JNK, sc-7345) or phosphorylated JNK (Thr183/Tyr185, sc-6254, all from Santa Cruz Biotechnology); mouse antibodies against β-actin (Sigma, A-1978) or KSHV ORF45 (Invitrogen, MA5-14769); mouse antibodies against KSHV ORF65 (a gift from Dr. Shou-Jiang Gao); and rat antibodies against HHV8 LANA (Abcam, Cambridge, United Kingdom, ab4103). Horseradish peroxidase (HRP)-conjugated goat anti-rabbit IgG (Bethyl, Montgomery, TX, United States, A120-101P), HRP-conjugated goat anti-mouse IgG (Bethyl, A90-101P), and HRP-conjugated goat anti-mouse IgG (Bethyl, A110-105P) were used as secondary antibodies. Protein bands were quantified by western blot analysis using Image Lab 6.0 software (Bio-Rad Laboratories).

### RNA Isolation, cDNA Synthesis, and Reverse Transcription-Quantitative Polymerase Chain Reaction Analysis

Total RNA was isolated from cultured cells using a Ribospin II RNA Isolation Kit (GeneAll Biotechnology, Seoul, South Korea). cDNA was synthesized using PrimeScript RT Master Mix (Takara, Shiga, Japan) at 37°C for 10 min. qPCR was performed using Takara TB Green FAST qPCR Mix (Takara). Cycling conditions were as follows: 95°C for 30 s, amplification for 40 cycles (95°C for 5 s and 60°C for 30 s), and melting curve analysis to confirm the specificity of the amplified product. All samples were run in triplicate, and gene expression levels were normalized to glyceraldehyde 3-phosphate dehydrogenase (GAPDH) expression. qPCR was performed and analyzed using a CFX96 real-time system (Bio-Rad Laboratories) and Bio-Rad CFX Manager software version 3.1 (Bio-Rad Laboratories). Data analysis was carried out as described previously (Yoo et al., 2005). qPCR was performed using the following primers: human GAPDH, sense (5′-GGTATCGTGGAAGGACTC-3′) and antisense (5′-GTAGAGGCAGGGATGATG-3′); KSHV LANA, sense (5′-TTGTGTATATGTGTATTGTCAGAA-3′) and antisense (5′-AACTTAACTATGGAAGATTGTAGG-3′); KSHV vCyclin, sense (5′-GCCTCACGCCTATTTCTA-3′) and antisense (5′-TTCTCCTGGTCTATAAGTTCTT-3′); KSHV ORF50, sense (5′-CGCTGTTGTCCAGTATTC-3′) and antisense (5′-AGAAGGTGACGGTATATCC-3′); KSHV K8, sense (5′-GCCTGCGTCTGTAGTTAA-3′) and antisense (5′-TCCTTATGTGCCTCCAATC-3′); KSHV ORF59, sense (5′-TTAGCCTGGAGTCCTTAATC-3′) and antisense (5′-GCA CACCTTCCACTTCTA-3′); KSHV ORF74, sense (5′-GCGATA GATATACTGCTCCT-3′) and antisense (5′-CATCAACACTTCT GCCAAT-3′); KSHV K8.1, sense (5′-TAAACCCACAGCCC ATAG-3′) and antisense (5′-CCACCACTACAACGACTA-3′); KSHV ORF65, sense (5′-ACTATCTCGTGTTCTTAATTGC-3′) and antisense (5′-ATGATCCCGCCTTTGAAT-3′). All primers were synthesized by Genotech (Daejeon, South Korea).

### Analysis of Kaposi’s Sarcoma-Associated Herpesvirus Genome Copy Number

For the relative quantification of intracellular KSHV genome DNA, KSHV-infected cells were lysed at 4 h of postinfection using a DNeasy Blood and Tissue kit (Qiagen, Hilden, Germany) according to the manufacturer’s recommendations. To analyze the virion copy number of the isolated KSHV, the supernatants of iSLK BAC16 cells with induction of lytic replication were collected and centrifuged at 100,000 × *g* for 1 h. The pellet was resuspended in 1X DNase buffer and then treated by RQ1 RNase-free DNase I (Promega, Madison, WI, United States) at 37°C for 1 h. DNA was extracted using the DNeasy Blood and Tissue kit (Qiagen). PCR analysis was carried out using the Takara TB Green FAST qPCR Mix (Takara, Shiga, Japan) with primers ORF26F (5′ GAC TCT TCG CTG ATG AAC TGG 3′) and ORF26R (5′ AGC ACT CGC AGG GCA GTA CG 3′) targeting KSHV ORF26 (Genotech, Daejun, South Korea). qPCR reaction conditions and data analysis followed the RT-qPCR as mentioned above. The number of viral DNA molecules was calculated from a standard curve constructed from serial dilutions of a known amount of pUC19 vector containing KSHV ORF26.

### Immunofluorescence Assay

Immunofluorescence assay was performed as described previously with minor modifications (Kang et al., 2021). Mouse antibodies against HHV-8 K8.1A/B (Santa Cruz Biotechnology, sc-65446) and KSHV ORF45 (Invitrogen, MA5-14769) were used as primary antibodies.

### Lactate Dehydrogenase Assay

Cytotoxicity was analyzed by a Cytotoxicity Detection kit PLUS (LDH, Roche, Basel, Switzerland) according to the manufacturer’s protocol. Cells were seeded at 3,000 cells/100 μl in a 96 well plate. The next day, SP600125 was treated with the indicated concentrations. After 24 h, the supernatant was transferred into the corresponding wells of a 96-well plate. The cultured cells with each condition were treated with the enclosed lysis solution for 15 min as a positive control. The dye solution-catalyst mixture was treated and incubated at room temperature for 10 min. The reaction was terminated with a stop solution, and absorbance was measured at 490 nm.

### Knockdown of C-Jun N-Terminal Kinase by Small Interfering RNA Transfection

Transfection of small interfering RNA (siRNA) was performed using Lipofectamine™ RNAiMAX Transfection Reagent (Thermo Fisher Scientific) according to the manufacturer’s instructions. Briefly, cells were seeded in 6-well plates with DMEM containing 10% FBS. The next day, the transfection reagent was diluted using Opti-MEM (Thermo Fisher Scientific). siRNA was treated at 100 μM. After 24 h post-transfection, cells were induced using sodium butyrate and Doxycycline. After 24 h post-transfection, cells were induced using sodium butyrate and Doxycycline. Predesigned siRNAs for JNK (MAPK8, 5599) and scrambled siRNA (AccuTarget negative control siRNA) were purchased from Bioneer Corporation (Daejeon, South Korea).

### Statistical Analysis

Results are shown as means ± standard deviations. The two-tailed Student’s t test was used to assess the significance of difference between groups. Statistical significance at *P* values of <0.05 and <0.01 is indicated by * and ^**^, respectively.

## Results

### Treatment With Dimethyl Sulfoxide During Lytic Replication Increased the Infectivity of Kaposi’s Sarcoma-Associated Herpesvirus Extracted From iSLK BAC16 Cells Through the Enhanced Production of Virions

**FIGURE 1:**
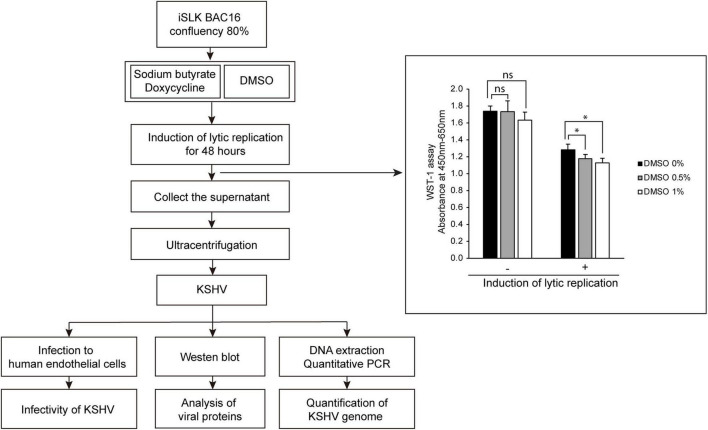
Schematic diagram of the experimental processes and cell viability of iSLK BAC16 cells by treatment of DMSO. Lytic replication was induced by sodium butyrate and doxycycline for 48 h. DMSO was added into culture media together with sodium butyrate and doxycycline at various concentrations. During lytic replication, cells were treated with various concentrations of DMSO to investigate its effects on virus production. After DMSO treatment, cell viabilities were analyzed by WST-1 assay. Data are shown as the mean ± SD, *n* = 3, ns, not significant; *p < 0.05. The produced KSHV was extracted by differential centrifugation and analyzed by infection of HUVECs, western blotting, and quantitative PCR (qPCR).

**FIGURE 2:**
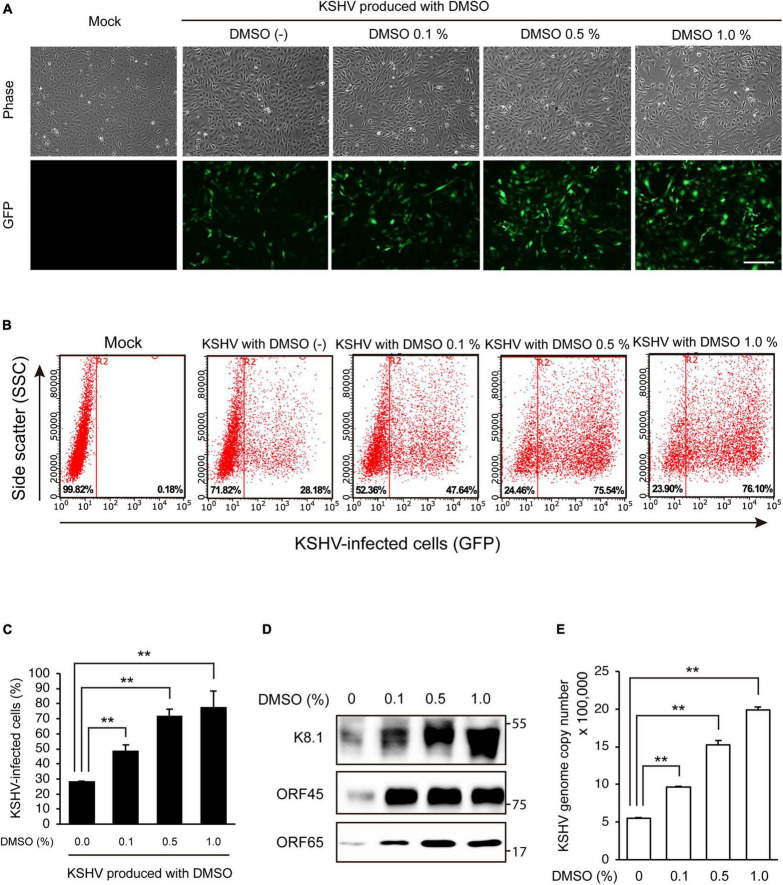
DMSO enhanced KSHV production in iSLK BAC16 cells. KSHV was harvested from iSLK BAC16 cells treated with various concentrations of DMSO during lytic replication. The same volume of KSHV from each group was used to infect HUVECs. **(A)** GFP expression of KSHV-infected cells in fluorescence microscopy. After 24 h of infection, infectivity was measured by analysis of GFP expression using fluorescence microscopy. Scale bar = 250 μm. **(B)** Representative results of the flow cytometric analysis of GFP expression in KSHV-infected HUVECs. KSHV-infected cells from panel **(A)** were detached and analyzed by flow cytometry. *X*- and *Y*-axis indicate GFP expression and side scatter, respectively. The numbers on the right lower corners of each panel indicate the percentage of GFP-positive cells. **(C)** The flow cytometric analysis of GFP expression in KSHV-infected HUVECs. Data are shown as the mean ± SD, *n* = 3, **p < 0.01. **(D)** Western blot analysis of KSHV proteins of the isolated KSHV from iSLK BAC16 cells. After induction of lytic replication, KSHV was extracted from the supernatants of iSLK BAC16 cells. iSLK BAC16 cells were treated with the indicated concentrations of DMSO during lytic replication. The same volume of extracted KSHV from each experimental condition was analyzed. **(E)** Analysis of KSHV genome copy numbers from the produced virions. Genomic DNA was isolated from the extracted virions and analyzed by qPCR with specific primers targeting KSHV *ORF26*. Data are shown as the mean ± SD, *n* = 3, **p < 0.01.

To investigate the effects of DMSO on KSHV-producing iSLK BAC16 cells, we extracted KSHV from iSLK BAC16 cells treated with various concentrations of DMSO during lytic replication and analyzed their infectivity in HUVECs. The schematic processes of virus production and analysis are presented in Figure 1. After treatment of DMSO to iSLK BAC16 cells, cell viability was analyzed (Figure 1). While DMSO alone did not affect cell viability, DMSO with inducing agents for lytic replication showed a significant decreased the cell viability with dose-dependent manners. HUVECs were infected with the same volume of extracted KSHV, and their infectivity was analyzed by assessment of GFP expression because this recombinant KSHV contained a GFP cassette. Interestingly, KSHV infectivity in HUVECs was increased for viruses harvested by treatment with DMSO compared with those without DMSO treatment (Figures 2A–C). In flow cytometry analysis, KSHV extracted from iSLK BAC16 cells treated with 1% DMSO showed an approximately 2.5-fold increase in infection rate compared with KSHV extracted from untreated cells (Figures 2B,C). These results indicated that KSHV extracted by adding DMSO resulted in a higher infection rate in HUVECs. A previous study reported that DMSO could affect protein structure and interaction (Chan et al., 2017). To investigate whether DMSO enhances infectivity of KSHV by modifying protein structures and receptor-ligand interaction, the isolated KSHV from iSLK BAC16 cells without DMSO treatment was infected with HUVECs together with DMSO (Supplementary Figure 1). Our results did not show any significant change of KSHV infectivity with DMSO treatment, suggesting that DMSO would not affect KSHV infectivity. To investigate whether the increase in KSHV infectivity was correlated with the increase in virion production, the extracted virions from each experimental group were analyzed by western blotting and qPCR. In western blot analysis, KSHV envelope protein K8.1, KSHV tegument protein ORF45, and KSHV capsid protein ORF65 were analyzed using specific antibodies (Figure 2D). The same volume of virus was extracted by adding various concentrations of DMSO, and the expression levels of all analyzed viral proteins were increased in proportion to DMSO. In addition, genomic DNA was extracted from KSHV virions, and KSHV genomic DNA was quantified using qPCR analysis of KSHV ORF26 (Figure 2E). KSHV genomic DNA was also increased by DMSO in a concentration-dependent manner. Our results indicated that the addition of DMSO enhanced KSHV virion production during the induction of lytic replication in KSHV iSLK BAC16 cells.

### Dimethyl Sulfoxide Enhanced Kaposi’s Sarcoma-Associated Herpesvirus Viral Gene Expression in iSLK BAC16 Cells During Lytic Replication

**FIGURE 3:**
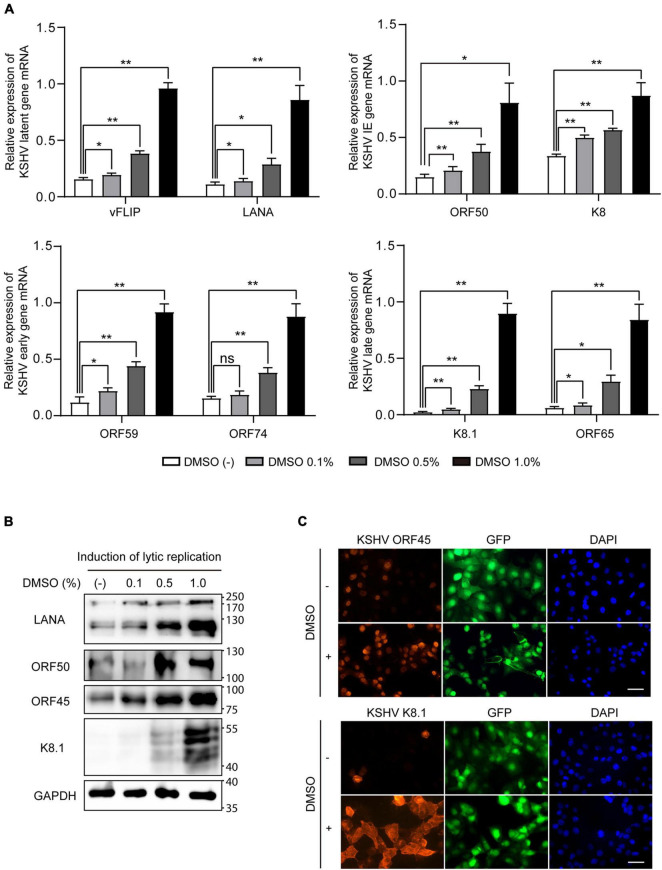
Increased expression of viral genes by DMSO during lytic replication of KSHV. iSLK BAC16 cells were treated with various concentrations of DMSO during lytic replication. After 24 h, total RNA and protein were isolated to analyze viral gene expression. **(A)** RT-qPCR analysis of mRNA expression of KSHV viral genes. Data are shown as the mean ± SD, *n* = 3, *p < 0.05, and **p < 0.01. **(B)** Western blot analysis of the indicated viral proteins in iSLK BAC16 with induction of lytic replication. **(C)** Immunofluorescence assay (IFA) for the indicated viral proteins in iSLK BAC16 with induction of lytic replication. DMSO (1%) was treated to cells at the beginning of lytic replication. IFA was analyzed at 24 h after induction. Scale bar = 50 μm.

**FIGURE 4:**
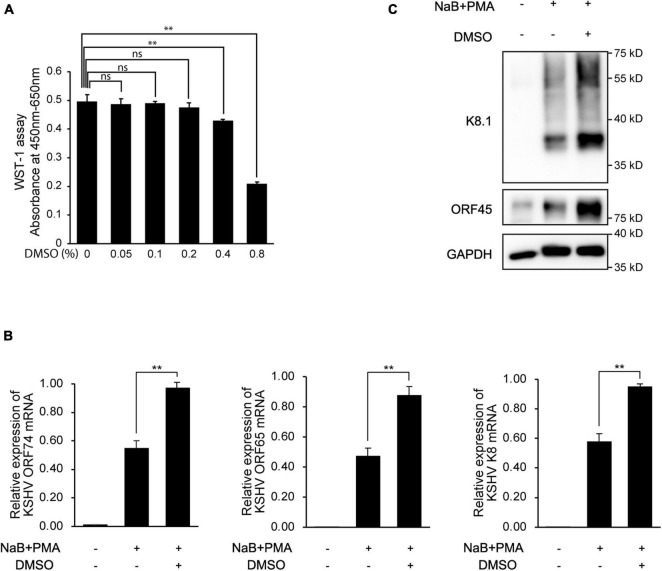
Enhanced expression of KSHV lytic genes in BCBL-1 cells by DMSO during reactivation. **(A)** Cell viability with various concentrations of DMSO. After 24 h of DMSO treatment, cell viabilities were analyzed by WST-1 assay. Data are shown as the mean ± SD, *n* = 3, ns, not significant; **p < 0.01. **(B)** RT-qPCR analysis of mRNA expression of KSHV viral genes. Reactivation of KSHV was induced in BCBL-1 by treatment with sodium butyrate (NaB, 0.3 mM) and phorbol-13-acetate (PMA, 20 ng/mL). DMSO (0.2%) was treated at the beginning of reactivation. RNA or protein lysate was extracted at 48 h after induction of lytic replication. Data are shown as the mean ± SD, *n* = 3, and **p < 0.01. **(C)** Western blot analysis of KSHV ORF45 and K8.1 in BCBL-1 cells.

Because viral production was increased by additional DMSO during lytic replication, we next evaluated whether DMSO-mediated virus production was correlated with lytic replication of KSHV. To this end, after treating iSLK BAC16 cells with various concentrations of DMSO for 24 h during lytic replication, total RNA, and cell lysates were extracted to analyze KSHV viral gene expression. During lytic replication, all analyzed mRNA expression of KSHV viral genes was significantly increased by DMSO treatment in a concentration-dependent manner (Figure 3A). Similarly, DMSO increased the expression of viral lytic proteins, such as ORF50, ORF45, and K8.1 in iSLK BAC16 cells under lytic replication (Figure 3B). Interestingly, mRNA and protein expression levels of latent genes, such as vFLIP (ORF71) and LANA (ORF73), were significantly increased by DMSO. Together, these results suggested that DMSO may affect not only lytic genes but also overall KSHV gene expression. To investigate whether DMSO increased the number of reactivating cells or increased copies of lytic gene transcripts within a reactivating cell, the expressions of KSHV lytic genes including ORF45 and K8.1 were analyzed by IFA (Figure 3C). The number of cells expressing ORF 45 and K8.1 was increased by DMSO treatment compared to the control group. These results indicated that DMSO could induce lytic replication in more cells. However, the possibility that DMSO increases gene expression in single cells cannot be completely excluded. To investigate DMSO also affects lytic replication of primary effusion lymphoma (PEL) cell line with KSHV, BCBL-1 was treated with a non-toxic concentration of DMSO (0.2%) during induction of lytic replication (Figure 4A). The lytic genes of KSHV were also enhanced by DMSO in BCBL-1 cells (Figures 4B,C), suggesting DMSO would have a similar stimulating effect on lytic replication on iSLK BAC16 cells and BCBL-1 cells.

### Dimethyl Sulfoxide Induced Faster Lytic Reactivation of Kaposi’s Sarcoma-Associated Herpesvirus in iSLK BAC16 Cells

**FIGURE 5:**
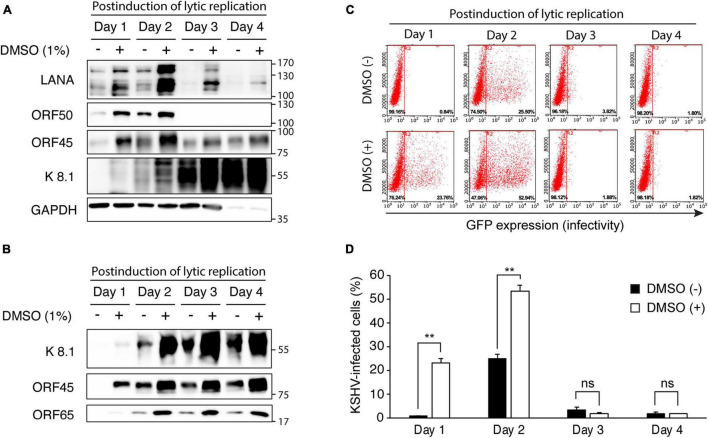
Time kinetic analysis of changes in viral gene expression and virus production of KSHV by DMSO treatment during reactivation of KSHV. **(A)** Western blot analysis for KSHV gene expression in iSLK BAC16 cells with DMSO. iSLK BAC16 cells were treated with DMSO and inducing agents for lytic replication together, then analyzed indicated viral proteins. **(B)** Western blot analysis of KSHV proteins of the isolated KSHV from iSLK BAC16 cells. After induction of lytic replication, KSHV was extracted from the supernatants of iSLK BAC16 cells at the indicated time points. The same volume of extracted KSHV from each experimental condition was analyzed. **(C,D)** The flow cytometric analysis of GFP expression in KSHV-infected HUVECs. The isolated KSHV from each condition infected with HUVECs, followed by analyzing GFP expression by flow cytometry at 24 h after infection. Representative results of the flow cytometric analysis of GFP expression in KSHV-infected HUVECs **(C)**. Analysis of KSHV infectivity **(D)**. Data are shown as the mean ± SD, *n* = 3, and **p < 0.01. ns, not significant.

Next, we analyzed time-kinetics for viral gene expression, virion production, and infectivity of produced KSHV to determine whether increased viral production with DMSO indeed increased viral production or simply rapidly induced reactivation of KSHV. In iSLK BAC16 cells with induction of lytic replication, we found that all analyzed viral proteins were increased in DMSO-treated cells than in no treated control (Figure 5A). Because we optimized our induction conditions for maximal virus production in a short time, most cells were dead after 2 days of induction. While the previous studies used doxycycline at 1 μg/mL and sodium butyrate at 1 mM (Brulois et al., 2012), we used doxycycline at 50 μg/mL and sodium butyrate at 1.2 mM for induction of lytic replication. Therefore, after 3 days of induction, most analyzed proteins except for the late protein K8.1 were decreased compared at 2 days of induction. In the extracted KSHV from the supernatant, all viral proteins from the DMSO-treated cells were also increased at all analyzed time points (Figure 5B). We found that the virus was produced from the cells treated with DMSO 1 day after induction of lytic replication, and the quantitative difference was also maintained until the fourth day. Therefore, it was confirmed that DMSO has the effect of not only enhancing KSHV production, but also induction of faster lytic replication and viral production (Figures 5A,B). To determine the infectivity of KSHV is proportional to the viral proteins of each extracted KSHV, the isolated KSHV was infected with HUVECs (Figures 5C,D). In the infected cells with virus extracted after induction of lytic replication for 1 day, GFP expression was not observed in the virus from the control cells not treated with DMSO. However, the KSHV from DMSO-treated cells showed high infectivity in HUVECs, which is consistent with the amount of virus protein analyzed in Figure 5B. At 2 days of induction, it was confirmed that the infection of the virus extracted from DMSO-treated cells was increased by about two times compared to the control. Regardless of DMSO treatment, the infectivity of the isolated KSHV was not observed after 3 days of induction of lytic replication. Since, in our experimental condition, most cells die after 2 days of induction, the virion may be inactivated in an acidic condition of culture media with dead cells.

### Dimethyl Sulfoxide Alone Did Not Induce Lytic Replication of Kaposi’s Sarcoma-Associated Herpesvirus in iSLK BAC16 Cells

**FIGURE 6:**
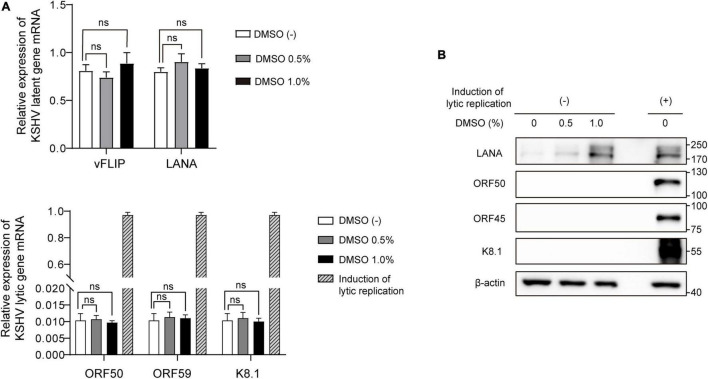
Differential expression of viral genes induced by DMSO during latent KSHV infection. iSLK BAC16 cells were treated with various concentrations of DMSO for 24 h. Next, total RNA and protein were isolated to analyze viral gene expression. **(A)** RT-qPCR analysis of mRNA expression of KSHV viral genes. Data are shown as the mean ± SD, *n* = 3, and ns, not significant. **(B)** Western blot analysis of the indicated viral proteins in iSLK BAC16 with (+) or without induction of lytic replication (–).

To explore the effects of DMSO on KSHV gene expression in latent infection, total RNA and cell lysates were extracted after treating iSLK BAC16 cells with various concentrations of DMSO for 24 h. During latent infection, we did not find any evidence that DMSO alone induced lytic replication without inducing reagents (Figure 6). mRNA expressions for latent and lytic genes were not affected by DMSO (Figure 6A). In protein levels, KSHV ORF50, ORF45, and K8.1 were not detected in DMSO treated iSLK BAC16 cells without induction of lytic replication (Figure 6B).

### Dimethyl Sulfoxide Enhanced the Lytic Replication Cycle by Induction of C-Jun N-Terminal Kinase Phosphorylation

**FIGURE 7:**
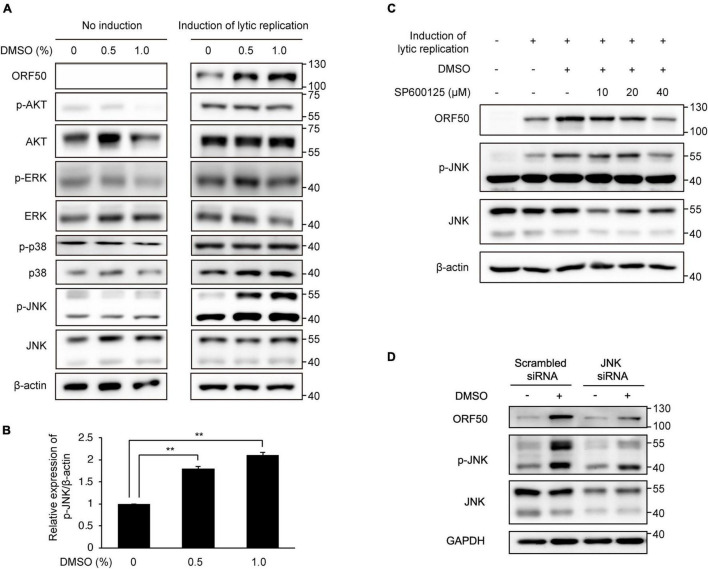
Enhanced lytic replication by DMSO was mediated by increased phosphorylation of JNK. Cells were then treated with DMSO during lytic replication. No induction samples were treated with DMSO alone. Lytic replication was induced in iSLK BAC16 cells with doxycycline and sodium butyrate. **(A)** Western blot analysis of signaling molecules related to lytic replication of KSHV. **(B)** Densitometric analysis of phospho-JNK/β-actin levels in iSLK BAC16 cells treated with DMSO and lytic replication-inducing agents. Data are shown as the mean ± SD, *n* = 3, and **p < 0.01. **(C,D)** Effects of the JNK inhibitor SP600125 or siRNA-mediated suppression of JNK on KSHV lytic replication in DMSO-treated iSLK BAC16 cells. Cells were treated with 10, 20, or 40 μM of SP600125 at the beginning of lytic activation with DMSO. After 24 h, cell lysates were collected, and the indicated proteins were analyzed by western blot analysis with specific antibodies **(C)**. For suppression of JNK with siRNA, iSLK BAC16 cells were transfected with JNK-specific small interfering RNA (JNK siRNA) and scrambled sequence control siRNA (scrambled siRNA) 24 h before lytic activation **(D)**.

Some cell signaling pathways, including the MAPK and phosphatidylinositol 3-kinase/AKT pathways, are known to be associated with KSHV lytic replication (Ford et al., 2006; Pan et al., 2006; Xie et al., 2008; Peng et al., 2010). Therefore, we next performed western blot analysis of several phosphorylated signaling proteins to investigate whether known signaling pathways related to KSHV lytic replication were associated with DMSO treatment in iSLK BAC16 cells under lytic replication. Although the levels of phospho-AKT, phospho-ERK, and phospho-p38 MAPK were not increased significantly by DMSO, phospho-JNK levels were increased in DMSO-treated iSLK BAC16 cells compared with that in untreated control cells (Figures 7A,B). In BCBL-1 cells, phospho-JNK was also increased by DMSO treatment during induction of lytic replication (Supplementary Figure 2). The JNK-specific inhibitor SP600125 was used to determine whether the enhancement of lytic replication in DMSO-treated cells was mediated by increased phosphorylation of JNK. Notably, at non-cell killing concentrations, SP600125 suppressed not only phospho-JNK levels but also KSHV ORF50 expression (Figure 7C and Supplementary Figure 3). To confirm the association of JNK and enhanced lytic replication by DMSO, siRNA for JNK was applied to iSLK BAC16 cells during lytic replication (Figure 7D). Suppression of JNK by siRNA attenuated ORF50 expression enhanced by DMSO treatment during lytic replication, which is consistent with that by the inhibition of JNK using SP600125 shown in Figure 7C. Taken together, our results suggested that JNK phosphorylation was associated with increased lytic replication induced by DMSO.

### Suppression of Dimethyl Sulfoxide-Mediated Enhancement of Kaposi’s Sarcoma-Associated Herpesvirus Production by Inhibition of C-Jun N-Terminal Kinase Pathway

**FIGURE 8:**
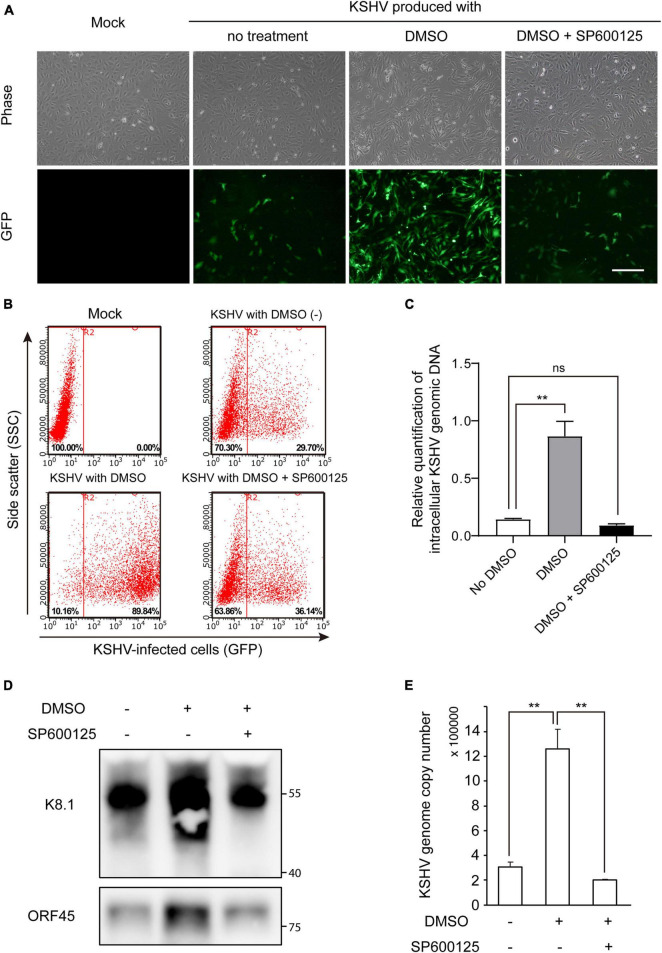
JNK inhibitor suppressed DMSO-mediated enhancement of KSHV virion production in iSLK BAC16 cells. iSLK BAC16 cells were treated with the JNK inhibitor SP600125 (20 μM) at the beginning of lytic activation with DMSO. After 2 days, KSHV was harvested from each culture supernatant. The same volume of KSHV from each group was used to infect HUVECs. **(A)** GFP expression of KSHV-infected cells, as evaluated by fluorescence microscopy. After 24 h of infection, GFP expression was observed using fluorescence microscopy. Scale bar = 250 μm. **(B)** Representative results of flow cytometric analysis of GFP expression in KSHV-infected HUVECs. GFP-expressing cells from panel **(A)** were detached and analyzed by flow cytometry. *X*- and *Y*-axis indicate GFP expression and side scatter, respectively. The numbers in the right lower corners of each panel indicate the percentage of GFP-positive cells. **(C)** qPCR for KSHV genomic DNA in KSHV-infected HUVECs. KSHV from each experimental group (no DMSO, DMSO alone, and DMSO with SP600125) was used to infect HUVECs, and genomic DNA was extracted at 4 h postinfection. Data are shown as the mean ± SD, *n* = 3, and ns, not significant. **p < 0.01. **(D,E)** Attenuated KSHV production by JNK inhibitor. After induction of lytic replication, KSHV was extracted from the supernatants of iSLK BAC16 cells. iSLK BAC16 cells were treated with DMSO or SP600125 during lytic replication. The same volume of extracted KSHV from each experimental condition was analyzed. Western blot analysis of KSHV proteins of the isolated KSHV virions **(D)**. Analysis of KSHV genome copy numbers from the produced virions **(E)**. Genomic DNA was isolated from the extracted virions and analyzed by qPCR with specific primers targeting KSHV *ORF26*. Data are shown as the mean ± SD, *n* = 3, and **p < 0.01.

Because JNK inhibition suppressed lytic replication of KSHV in DMSO-treated iSLK BAC16 cells (Figure 7C), we investigated whether JNK inhibitor, SP600125, also suppressed DMSO-mediated enhancement of KSHV production. After isolation of KSHV from iSLK BAC16 cells, HUVECs were infected with the virus. As shown in Figures 8A,B, GFP-expressing cells were prominent in DMSO-treated KSHV-infected cells. KSHV produced from iSLK BAC16 cells treated with both DMSO and SP600125 showed less infectivity than KSHV produced with DMSO alone (Figure 8B and Supplementary Figure 4), suggesting that the JNK inhibitor suppressed the DMSO-mediated enhancement of KSHV production in iSLK BAC16 cells. KSHV genomic DNA in KSHV-infected HUVECs was significantly suppressed following infection of cells with KSHV extracted from DMSO- and SP600125-treated iSLK BAC16 cells compared with that in cells treated with DMSO alone (Figure 8C), consistent with the results shown in Figures 8A,B. As a result of analyzing KSHV isolated from the supernatant under each condition, the virus proteins and KSHV genome copy number were suppressed in the SP600125-treated group (Figures 8D,E) and siRNA for JNK-transfected group (Supplementary Figure 5), suggesting that DMSO-mediated enhancement of KSHV production would be associated with the JNK pathway.

## Discussion

We found that DMSO enhanced KSHV lytic gene expression, resulting in at least a twofold increase in KSHV virion production. During lytic replication, DMSO increased both viral mRNA and protein expression. Although KSHV LANA protein was upregulated by DMSO, even during latent infection, DMSO alone did not induce lytic replication under our experimental conditions. We also demonstrated that the phosphorylation of JNK was associated with enhanced lytic replication and KSHV production induced by DMSO.

Although DMSO is the most commonly used solvent for cryopreservation and *in vitro* assays, its biological effects are frequently overlooked. A recent study showed that DMSO significantly affects not only the expression of mRNA/microRNA but also DNA methylation in three-dimensional cardiac and hepatic microtissues (Verheijen et al., 2019). Several studies have reported that DMSO affects the life cycles of viruses. For example, the replication or virus of fowl plague virus (Scholtissek and Muller, 1988), human immunodeficiency virus 1 (Taddeo et al., 2000), and duck hepatitis B virus (Tsuei et al., 1979) is increased by DMSO. In addition, DMSO induces reactivation of murine cytomegalovirus in latently infected mouse spleens (Blackett et al., 1987).

In many previous studies on KSHV, DMSO has been frequently used *in vitro* studies as a vehicle for chemical agents (Lee et al., 2014; Jeon et al., 2017; Kang et al., 2021). Intriguingly, we found that DMSO enhanced the expression of KSHV viral proteins, including LANA, ORF50, ORF45, and K8.1, during lytic replication. The phosphorylation of JNK was found to be associated with DMSO-mediated enhancement of lytic replication of KSHV because JNK phosphorylation was increased as ORF50 expression was increased by DMSO, whereas inhibition of JNK phosphorylation suppressed both lytic replication and KSHV production. A previous study demonstrated that DMSO induced JNK phosphorylation in human endothelial cells (Liang et al., 2011). Indeed, DMSO is known to have diverse biological effects on cells. Therefore, we could not exclude the possibility that another mechanism may be associated with the enhanced lytic replication of KSHV by DMSO.

In latent infection, the expression of KSHV LANA protein was also increased by treatment with DMSO. However, unlike the results in lytic replication, wherein both LANA RNA and protein were upregulated by DMSO, we did not observe increased mRNA levels during latent infection. These results revealed that DMSO affected viral gene expression during both latent and lytic replication of KSHV; however, the underlying mechanisms may be different in latent and lytic replication. Since DMSO did not increase transcript levels for latent genes in the same condition, DMSO may regulate LANA expression in the translation or protein degradation process. Further studies are required to evaluate the exact mechanisms through which DMSO regulates LANA expression in latent KSHV infection.

In summary, this is the first study showing that DMSO enhanced lytic replication of KSHV and virion production during induced lytic replication. We showed that DMSO effectively increased KSHV production in the KSHV-producing cell line iSLK BAC16. Our findings may be helpful for studies requiring larger amounts of KSHV virions.

## Data Availability Statement

The original contributions presented in the study are included in the article/Supplementary Material, further inquiries can be directed to the corresponding author.

## Author Contributions

S-KK and M-SL designed the study. S-KK, M-JL, JL, and H-HR performed the experiments and analyzed the data. S-KK, H-HR, and M-SL wrote the manuscript. All authors read and approved the final manuscript.

## Conflict of Interest

The authors declare that the research was conducted in the absence of any commercial or financial relationships that could be construed as a potential conflict of interest.

## Publisher’s Note

All claims expressed in this article are solely those of the authors and do not necessarily represent those of their affiliated organizations, or those of the publisher, the editors and the reviewers. Any product that may be evaluated in this article, or claim that may be made by its manufacturer, is not guaranteed or endorsed by the publisher.
