# Supplementary material for: Dimethyl Sulfoxide Enhances Kaposi’s Sarcoma-Associated Herpesvirus Production During Lytic Replication
Source: Front Microbiol. 2021 Dec 16;12:778525. doi: 10.3389/fmicb.2021.778525 (PMC8716793; doi:10.3389/fmicb.2021.778525)
Supplement: Supplementary file 1 [file Data_Sheet_1.PDF]

Supplementary Material

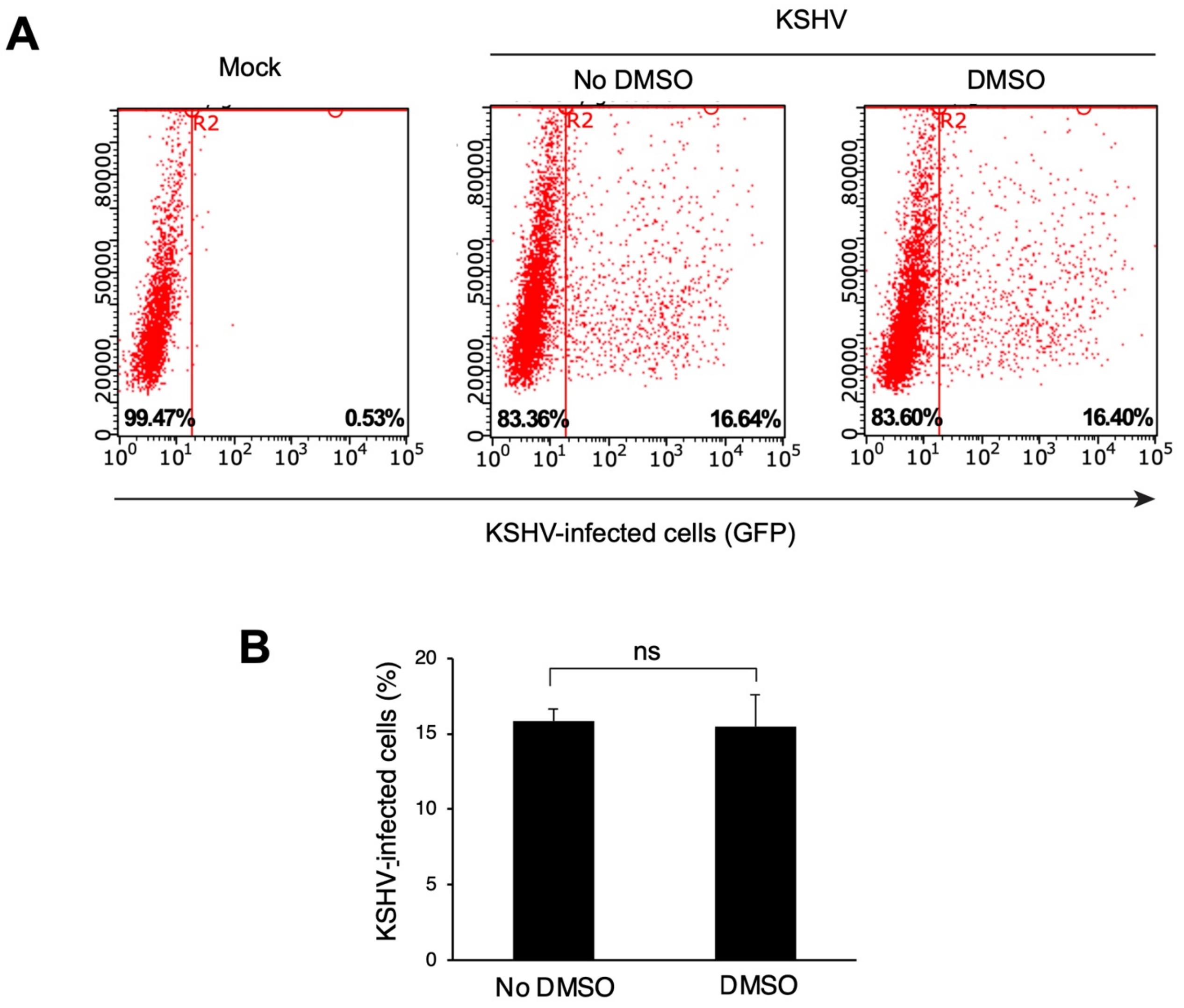

Supplementary Figure 1. Analysis of KSHV infectivity in HUVECs with DMSO treatment during infection. KSHV isolated from iSLK BAC16 cells was infected with HUVECs with or without DMSO treatment (1%). After 24 h after infection, KSHV infectivity was measured by GFP expression using flow cytometric analysis. (A) A representative flow cytometric analysis for KSHV infectivity. (B) Analysis of KSHV infectivity in HUVECs. Data are shown as the mean ± SD, n = 3, ns: not significant.

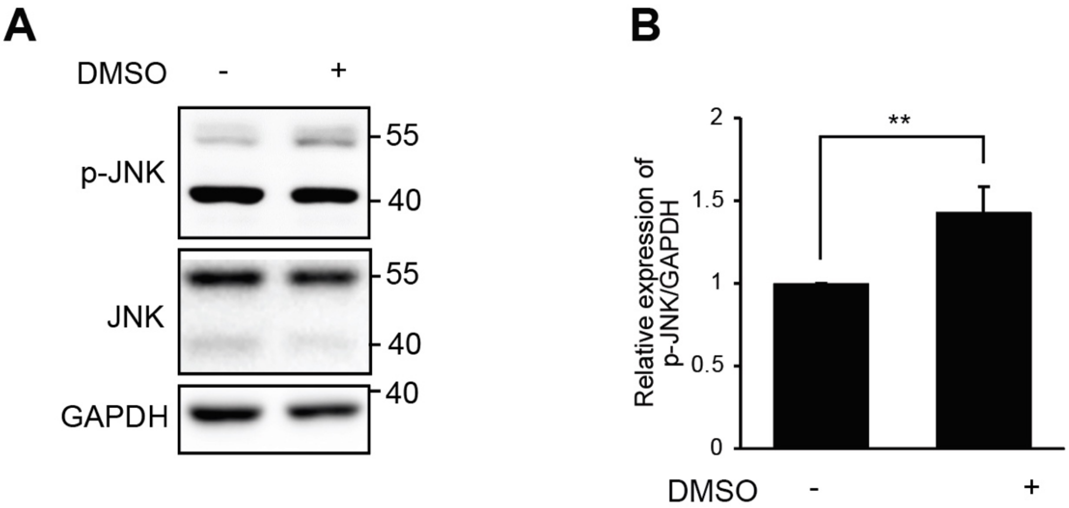

Supplementary Figure 2. Enhanced expression of phosphorylation of JNK in BCBL-1 cells by DMSO during reactivation. (A) Western blot analysis of JNK and p-JNK in BCBL-1 cells. Reactivation of KSHV was induced in BCBL-1 by treatment with sodium butyrate (NaB, 0.3 mM) and phorbol-13-acetate (PMA, 20 ng/mL). DMSO was treated at the beginning of reactivation. Protein lysate was extracted at 48 h after induction of lytic replication. (B) Densitometric analysis of phospho-JNK/GAPDH levels in BCBL-1 cells treated with DMSO and lytic replication-inducing agents. Data are shown as the mean ± SD, n = 3, $^{**}p < 0.01$.

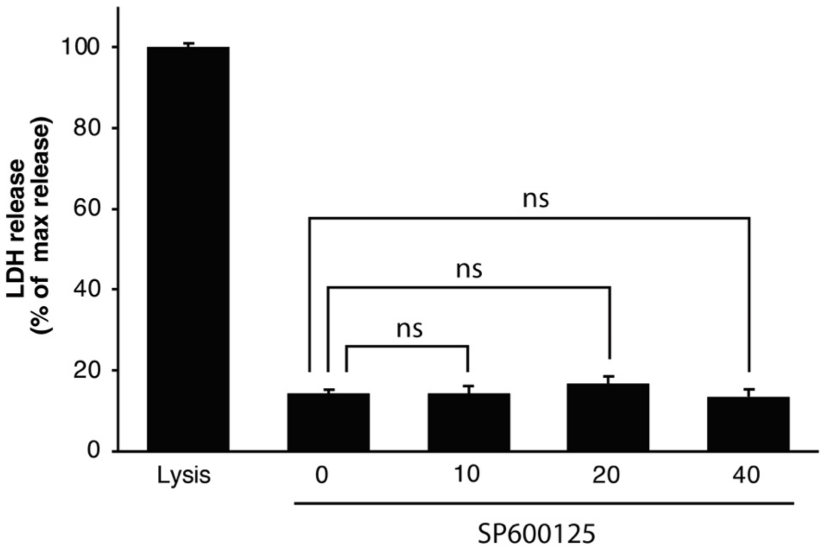

Supplementary Figure 3. Cell death analysis in iSLK BAC16 cells with SP600125. Each concentration of SP600125 was treated to iSLK BAC16 cells for 24 h. Then cell death was analyzed by LDH assay. Lysis represents 100% of LDH release. Data are expressed as a percentage of the samples treated with lysis solution (Lysis). Data are shown as the mean ± SD, n = 3, ns: not significant.

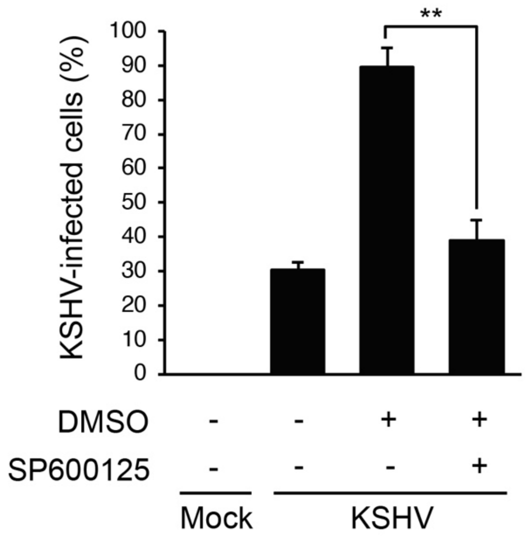

Supplementary Figure 4. JNK inhibitor suppressed DMSO-mediated enhancement of KSHV virion produced in iSLK BAC16 cells. KSHV was isolated from the three different experimental conditions, no treatment control, DMSO alone, and DMSO mixed with JNK inhibitor SP600125. Then, the same volume of isolated viruses from each group was used to infect HUVECs. The flow cytometric analysis of GFP expression in KSHV-infected HUVECs at 24 h of postinfection. Data are shown as the mean ± SD, n = 3, **$p < 0.01$.

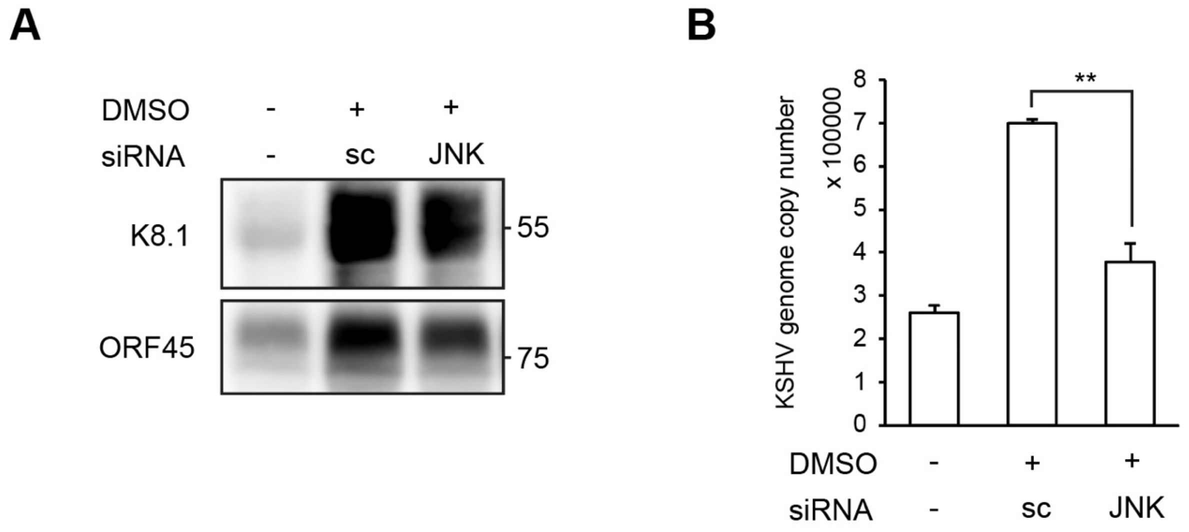

Supplementary Figure 5. siRNA for JNK suppressed DMSO-mediated enhancement of KSHV virion production. iSLK BAC16 cells were transfected with scrambled siRNA control (sc) or siRNA for JNK (JNK). After 24 h, lytic replication was induced by inducing agents together with DMSO. After two days, KSHV was harvested from each culture supernatant. The same volume of KSHV from each group was used for DNA or protein extraction. (A) Western blot analysis of KSHV proteins of the isolated KSHV virions. (B) Genomic DNA was isolated from the extracted virions and analyzed by qPCR with specific primers targeting KSHV *ORF26*. Data are shown as the mean ± SD, n = 3, **$p < 0.01$.
